# The Prognostic Value of Whole-Blood PSMB5, CXCR4, POMP, and RPL5 mRNA Expression in Patients with Multiple Myeloma Treated with Bortezomib

**DOI:** 10.3390/cancers13050951

**Published:** 2021-02-25

**Authors:** Pawel Robak, Dariusz Jarych, Damian Mikulski, Izabela Dróżdż, Edyta Węgłowska, Aleksandra Kotkowska, Małgorzata Misiewicz, Piotr Smolewski, Konrad Stawiski, Wojciech Fendler, Janusz Szemraj, Tadeusz Robak

**Affiliations:** 1 Department of Experimental Hematology, Medical University of Lodz, 93-510 Lodz, Poland; pawel.robak@umed.lodz.pl (P.R.); piotr.smolewski@umed.lodz.pl (P.S.); 2 Laboratory of Personalized Medicine, Bionanopark, 93-465 Lodz, Poland; djarych@cbm.pan.pl (D.J.); e.weglowska@bionanopark.pl (E.W.); 3 Laboratory of Virology, Institute of Medical Biology, Polish Academy of Sciences, 93–232 Lodz, Poland; 4 Department of Biostatistics and Translational Medicine, Medical University of Lodz, 92-215 Lodz, Poland; damian.mikulski@stud.umed.lodz.pl (D.M.); konrad.stawiski@umed.lodz.pl (K.S.); wojciech_fendler@dfci.harvard.edu (W.F.); 5 Department of Clinical Genetics, Medical University of Lodz, 92-213 Lodz, Poland; izabela.drozdz@umed.lodz.pl; 6 Department of Hematology, Medical University of Lodz, 93-510 Lodz, Poland; aleksandra.kotkowska@umed.lodz.pl (A.K.); malgorzata.misiewicz@umed.lodz.pl (M.M.); 7 Department of Medical Biochemistry, Medical University of Lodz, 92-215 Lodz, Poland; janusz.szemraj@umed.lodz.pl

**Keywords:** bortezomib, CXCR4, gene expression, multiple myeloma, POMP, PSMB5, refractory, RPL5, TXN, XBP1

## Abstract

**Simple Summary:**

The mRNA expression of nine previously described genes that may affect resistance to multiple myeloma (MM), viz., *ABCB1*, *CXCR4*, *MAF*, *MARCKS*, *POMP*, *PSMB5*, *RPL5*, *TXN,* and *XBP1*, was compared between bortezomib-refractory and bortezomib-sensitive patients. *RPL5* was the only gene to be significantly down-regulated in MM patients compared with non-MM individuals, while *POMP* was significantly up-regulated in the bortezomib-refractory patients. Multivariate analysis found the best independent predictors of progression-free survival to be high *PSMB5* and *CXCR* expression and autologous stem cell transplantation, and that high expression of *POMP* and *RPL5* were associated with shorter survival.

**Abstract:**

Proteasome inhibitors, like bortezomib, play a key role in the treatment of multiple myeloma (MM); however, most patients eventually relapse and eventually show multiple drug resistance, and the molecular mechanisms of this resistance remain unclear. The aim of our study is to assess the expression of previously described genes that may influence the resistance to bortezomib treatment at the mRNA level (*ABCB1*, *CXCR4*, *MAF*, *MARCKS*, *POMP*, *PSMB5*, *RPL5*, *TXN,* and *XBP1*) and prognosis of MM patients. mRNA expression was determined in 73 MM patients treated with bortezomib-based regimens (30 bortzomib-sensitive and 43 bortezomib-refractory patients) and 11 healthy controls. *RPL5* was significantly down-regulated in multiple myeloma patients as compared with healthy controls. Moreover, POMP was significantly up-regulated in MM patients refractory to bortezomib-based treatment. In multivariate analysis, high expression of *PSMB5* and *CXCR* and autologous stem cell transplantation were independent predictors of progression-free survival, and high expression of *POMP* and *RPL5* was associated with shorter overall survival.

## 1. Introduction

Multiple myeloma (MM, plasma cell myeloma) is a hematological malignancy characterized by the accumulation of malignant plasma cells (PC) in the bone marrow (BM), often resulting in bone lesions, hypercalcemia, infections, anemia, and production of monoclonal immunoglobulin [1]. The disease occurs mainly in older patients and accounts for 15% of all hematologic malignancies, with an annual incidence of 4.5–6 cases per 100,000 [2], with an estimated 32,270 new cases and 12,830 deaths in the United States in 2020 [3]. Proteasome inhibitors (PI) play a key role in the treatment of MM [4,5,6]. Three PIs, bortezomib, carfilzomib, and ixazomib, are currently approved for the treatment of MM and several others are undergoing clinical trials [7].

Bortezomib is the first-in-class selective and reversible inhibitor of the 26S proteasome. It demonstrates antiproliferative and antitumor activity, and its use has been a breakthrough in treating MM in the past 15 years [5]. It is a boronic acid-based compound, which inhibits β5 chymotrypsin-like (CT-L) and to a lesser extent, β1 caspase-like (C-L) of the proteasome; it has been approved for treatment both in front-line and in relapsed/refractory patients [7]. However, the development of resistance and side effects can limit its use in MM [8]. Most patients show resistance to bortezomib after several courses of treatment and most of them demonstrate multiple drug resistance. In addition, approximately 20% of patients exhibit primary resistance, which determines lack of response to treatment [8,9].

Although resistance to PIs appears to be acquired through a number of different mechanisms, genetic abnormalities play a key role for most anti-myeloma drugs [8,10,11,12]. Single-point mutations and modification of gene expression in neoplastic cells refractory to PI have been reported in previous studies [11,13,14,15,16]. Several genes associated with bortezomib resistance have been identified in MM cells, including *POMP*, *XBP1*, *PSMB5*, *MARCKS*, *ABCB1*, *CXCR4*, *MAF*, *TXN*, *TJP1*, *RPL5*, *CDK5,* and *CYP1A1* [16,17,18,19,20,21,22,23]; however, these genes have been examined individually, and usually only using commercially available MM cell lines. The aim of our study was to evaluate the prognostic value of nine previously described genes that may affect the prognosis in patients with a clinically detected loss of response to bortezomib treatment: *ABCB1*, *CXCR4*, *MAF*, *MARCKS*, *POMP*, *PSMB5*, *RPL5*, *TXN,* and *XBP1*. A better understanding of the genetic disorders involved in MM drug resistance can improve the prognosis and prognostication, and assist the development of new therapeutic options to improve the treatment of this disease.

## 2. Results

The demographic, clinical, and laboratory characteristics of the MM patients enrolled for the study are presented in Table 1. Overall, 30 of the 73 patients were bortezomib sensitive, while the other 43 were refractory. No statistically significant differences were observed between bortezomib-sensitive and bortezomib-refractory MM patients with regard to bone involvement at diagnosis (*p* = 0.96), calcium > 2.75 mmol/L at diagnosis (*p* = 0.89), creatinine > 2 mg/dL at diagnosis (*p* = 0.31) or Hb < 10 g/dL at diagnosis (*p* = 0.73) and ISS (*p* = 0.86). The only statistically significant difference was observed in predominant paraprotein level (*p* = 0.02). In addition, light chain disease (LCD) was more common (36.7%) among the sensitive group than the refractory group (9.3%).

Twelve patients had received at least one prior therapy before bortezomib-based regimen initiation and 11 of them had become refractory to bortezomib. It was found that 41 patients displayed IgG paraprotein, 17 demonstrated IgA, and 15 had LCD. Most of the patients (79.5%) had received a bortezomib, cyclophosphamide, and dexamethasone (VCD) regimen, six (8.2%) VMP (bortezomib, melphalan, and prednisone), four (5.5%) VTD (bortezomib, thalidomide, and dexamethasone), another four VD (bortezomib and dexamethasone), and one received IsaVRd (isatuximab, lenalidomide, bortezomib, and dexamethasone). Cytogenetics data were available for 41 patients (56.1%). Amp (1q) was the most common abnormality (53.7%), followed by IGH rearrangements (46.3%), t(4;14) (22.0%), and del(13q) (19.5%).

**Figure 1:**
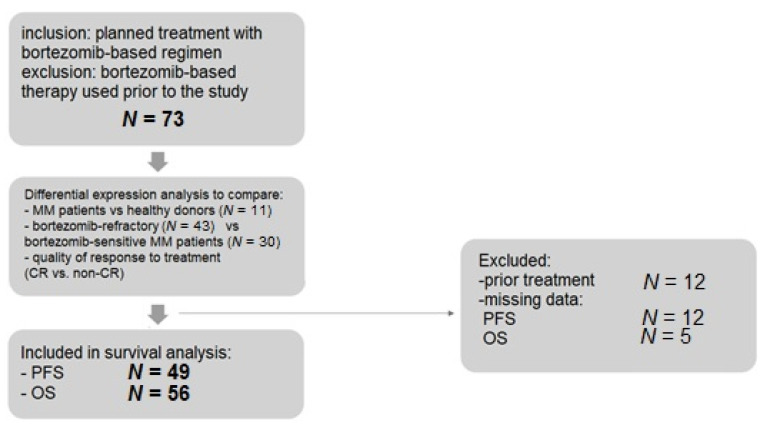
Flowchart of study protocol and main analyses. Abbreviations: CR—complete response; MM—multiple myeloma; OS—overall survival; PFS—progression-free survival.

**Figure 2:**
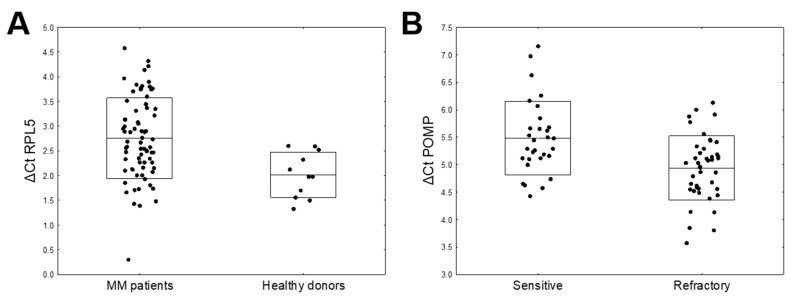
Dot plot representation of the ΔCt values of differentially expressed mRNA. The box plots depict the mean and SD. A higher ΔCt value represents the lower expression of the gene at the mRNA level: (**A**) ΔCt of *RPL5* in multiple myeloma patients healthy donors (*p* = 0.0033) and (**B**) ΔCt of *POMP* in sensitive and refractory to bortezomib MM patients (0.0062).

A flowchart depicting the number of patients in all stages of the study, and giving reasons for exclusion, is presented in Figure 1. The expression of nine mRNAs (*ABCB1*, *CXCR4*, *MAF*, *MARCKS*, *POMP*, *PSMB5*, *RPL5*, *TXN,* and *XBP1*) was determined in all 73 MM patients treated with bortezomib-based regimens and the 11 non-MM controls. Differential expression analysis indicated that *RPL5* was significantly down-regulated in MM patients compared with controls (Table 2, Figure 2A). Moreover, *POMP* was significantly up-regulated in bortezomib-refractory MM patients (Table 3, Figure 2B). No statistically significant differences were found between the groups with regard to the expression of selected mRNAs and the quality of response to treatment (Tables S1 and S2).

To provide a unified assessment of the prognostic impact of selected mRNA expression level at diagnosis, twelve patients who had received prior treatment before the bortezomib-based regimen were excluded from the outcome analysis. In the course of multiple myeloma, the duration of response decreases consistently with each successive regimen [24]. In this way, previous treatment itself is a factor that severely impacts PFS. Data on PFS was available in 11/12 previously treated patients, and the impact of this factor is presented in Figure S1. In contrast, in the previously treated group, no statistically significant differences were observed in mRNA expression (Table S3); however, in order to increase the statistical power of the analysis, this group was not excluded from differential expression analyses.

**Figure 3:**
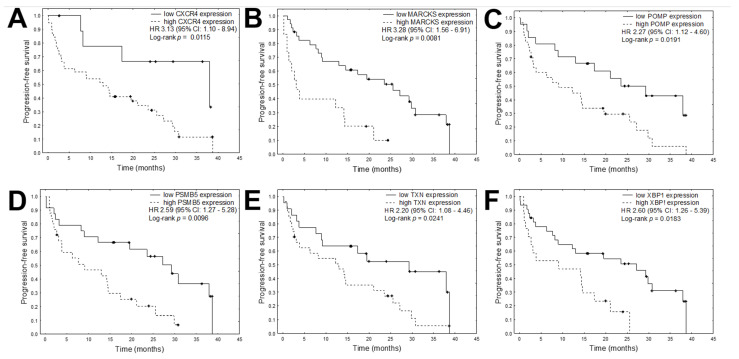
Kaplan–Meier plots for each of the significant mRNAs in the univariate analyses for PFS: (**A**) *CXCR4*, (**B**) *MARCKS*, (**C**) *POMP*, (**D**) *PSMB5*, (**E**) *TXN*, (**F**) *XBP1*.

**Figure 4:**
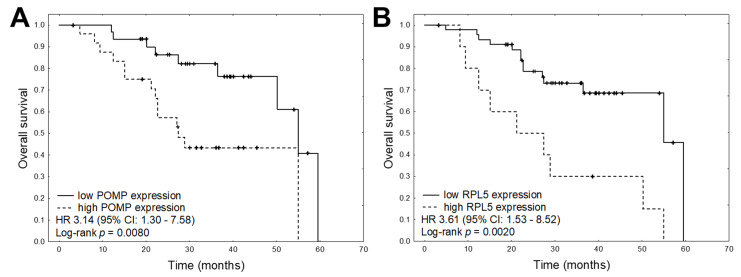
Kaplan–Meier plots for each of the significant mRNAs in the univariate analyses for OS: (**A**) *POMP*, (**B**) *RPL5.*

**Figure 5:**
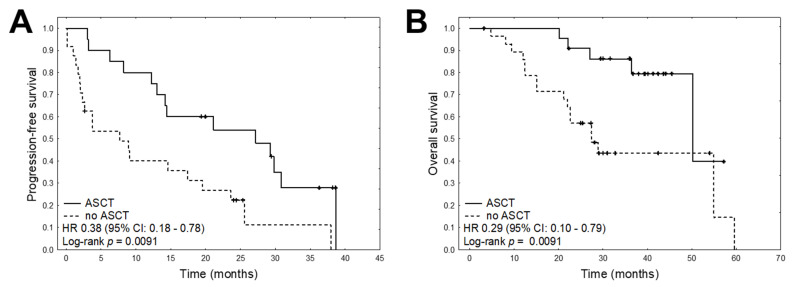
Kaplan–Meier plots for ASCT in the univariate analyses for (**A**) PFS and (**B**) OS.

Overall, data on progression free survival (PFS) were available for 49 patients and data on overall survival (OS) for 56 patients. The median PFS was 14.4 months and the median OS was 29.0 months. Univariate Cox proportional hazards regression analysis was conducted to determine the prognostic value of the quantified mRNA expression; the results indicated that in MM patients, higher expression of *CXCR4*, *MARCKS*, *POMP*, *PSMB5*, *TXN,* and *XBP1* was significantly correlated with shorter PFS (Table 4, Figure 3). Univariate analysis found higher expression of *POMP* and *RPL5* to be associated with shorter OS in MM patients (Figure 4). In addition, the only clinical variable that was related to PFS and OS was the use of autologous stem cell transplantation (ASCT) during the treatment schedule (Figure 5).

The PFS analyses included 12 cases of missing data. Therefore, to check its robustness, we repeated the univariate Cox regressions, including seven of the missing cases for which OS time was known. The analysis yielded similar hazard ratios and *p*-values as before, and the previously significant mRNAs maintained their significance (Table S4).

To further investigate the prognostic factors, multivariate analyses were carried out using Cox’s proportional hazards regression model with a stepwise selection procedure. As ASCT was the only significant clinical variable in our univariate analyses with proven prognostic significance, it was entered as covariate in the multivariable model. The results found high expression of *PSMB5* and *CXCR* and the presence of ASCT to be the best independent predictors of PFS (Table 5). Multivariate analysis of OS found high expression of *POMP* and *RPL5* to be associated with shorter survival.

We repeated our analyses with mRNA expression as a continuous variable (Table S5). In the univariate analyses, *PSMB5* and *CXCR4* lost their significance. In the next step, multivariate proportional hazard regression was performed with a stepwise selection procedure (Model 2, Table S6). An approach based on dichotomized variables yielded a model with a better fit to the data and with a lower AIC value.

## 3. Discussion

The study comprehensively determined the mRNA expression of nine genes that may affect resistance in 73 MM patients treated with bortezomib-based regimens and 11 healthy volunteers: *ABCB1*, *CXCR4*, *MAF*, *MARCKS*, *POMP*, *PSMB5*, *RPL5*, *TXN,* and *XBP1*. The genes were selected on the basis of previous laboratory and clinical studies investigating the bortezomib resistance in MM patients [17,18,19,20,21,25]. For the present study, it was decided to evaluate the gene expression using whole-blood samples, as this is an easier procedure to perform in clinical practice than preliminary PC isolation. However, standardization of mRNA expression profiling after cytometric isolation of specific population of cells may be technically challenging because of the variability of material quality, cell number, and other factors important at this experimental scale (cell cycle, mutation profile, clonicity etc.). Moreover, designing a model based only on a selected population of cells could downplay the interactions between the cells and the immune system, as well as other unforeseeable effects. Restricting the analysis to a cell subset would therefore potentially result in a potentially, very accurate test if cells are isolated correctly, but not sufficiently robust to use in different settings, with different technical tools and in varying clinical scenarios. Although a functional analysis of how these genes change their activity within cells during different phases of treatment would be an exciting study to perform, it would likely require a different experimental model, cell cultures, and in-depth mechanistic evaluations far exceeding the scope of this survival-oriented analysis. A similar method but based only on leukocytes in peripheral blood, not whole blood, was recently used by Watanabe et al. in evaluating the novel biomarkers to predict bortezomib response in MM patients [26].

According to the differential expression analysis, *RPL5* gene was the only gene that was significantly down-regulated in MM patients compared to the normal individuals; however, higher *RPL5* expression correlated with shorter survival in MM patients. *RPL5* has also been found to be deleted in 20–40% of MM patients, and it is the only recurrently mutated ribosomal protein gene in MM [27,28].

In addition, *RPL5* mRNA expression level was proposed as a clinical biomarker for response to bortezomib in MM patients; Hofman et al. [28] reported significantly lower *RPL5* mRNA expression in patients with MM who initially responded to bortezomib and then relapsed, and both newly diagnosed and relapsed patients with low *RPL5* expression had better PFS when bortezomib was used in their treatment. In addition, they reported an association between low *RPL5* mRNA levels and initial response to bortezomib in relapsed MM patients. *RPL5* expression has also been associated with shorter survival in newly diagnosed patients [28].

In our study, *POMP* gene was significantly up-regulated in MM patients refractory to bortezomib-based treatment in comparison with bortezomib-sensitive patients. Higher expression of *POMP* was found to be associated with shorter survival: POMP protein expression is essential for the biogenesis of proteasome de novo and its increased expression facilitates acquired resistance to PI [16]. An increase in POMP protein expression has also been noted in V10R, RPMI 8226, OPM-2, ANBL-6, and KAS-6/1 MM cells resistant to bortezomib [16,29]. Similarly to the present study, POMP protein suppression via shRNAs restored cell sensitivity, while over-expression favored resistance.

A protein-binding site for a suppressive factor, NRF2, has also been identified in the promoter region of the POMP protein. Although its increased expression should increase sensitivity to bortezomib, expression of POMP has been found to be increased in resistant cells, together with increased levels of POMP protein. The activation of both proteins varies according to cell line, and POMP appeared to have a greater effect on bortezomib sensitivity in the KAS-6/1 than OPM-2 line [30].

In the MM patients in the present study, univariate Cox proportional hazards regression analysis found the expression of six of the nine studies genes, viz. *PSMB5*, *CXCR4*, *MARCKS*, *POMP*, *TXN,* and *XBP1*, to significantly correlate with PFS. In addition, the multivariate analysis found high expression of *PSMB5*, *CXCR,* and *ASCT* to be the best independent predictors of PFS. Proteasome subunit β type 5 (PSMB5) is the target for bortezomib and other PI inhibitors that harbor chymotrypsin-like proteolytic activity [31]. Bortezomib occupies the PSMB5 substrate-binding pocket, interfering with the catalytic N-terminal threonine residue. Apart from β5 point mutations, the most frequent change observed in the bortezomib-resistant cell lines was overexpression of the β5 subunit [21,32,33,34]. A recent study by Barrio et al. identified somatic PSMB5 substitutions in an MM patient treated with bortezomib, suggesting that resistance acquired through PSMB5 point mutations is clinically relevant [21]. Recently, in KMS-18 and KMS-27 MM cells, the PSMB5 gene was found to harbor novel bortezomib resistance alleles which determine response to second-generation proteasome inhibitors in MM [35]. In addition, PSMB5 deletion resensitized drug-resistant, PSMB5-mutated cell lines to bortezomib, suggesting that PSMB5 mutation plays a role in drug resistance [36].

Our findings indicated that higher *CXCR4* expression correlated with shorter PFS. CXCR4 is a pleiotropic chemokine receptor which acts through its ligand (CXCL12) and influences proliferation, invasion, dissemination, and drug resistance in MM [37,38]. The current therapeutic focus is on disrupting the interaction of MM cells with their protective tumor microenvironment, in which the CXCR4 axis plays an essential role [39]. In contrast to our present study, reduced expression of CXCR4, a single biomarker in the Bcl-XL/Myc model system, has indicated poorer outcomes in MM patients treated with bortezomib [40]. In addition, low CXCR4 expression was associated with a worse outcome than high CXCR4 expression, and correlated with increased MM severity and aggressiveness in patients treated with bortezomib, either alone or in combination with other agents [18,40].

The univariate Cox proportional hazards regression analysis found that the higher expression of *MARCKS*, *TXN,* and *XBP1* significantly correlated with shorter PFS in MM patients. Another marker of PI resistance is MARCKS. This protein is important in cell adhesion and metastatic spread [41] and is involved in resistance to apoptosis in prostate cancer cells [16]. Its expression is significantly elevated in many types of cancer [42]. Micallef et al. reported overexpression of MARCKS in nine of 18 (50%) studied MM cell lines [43]; in addition, in line with our present findings, Yang et al. reported increased MARCKS expression in bortezomib-refractory MM patients, as well as increased bortezomib sensitivity in bortezomib-resistant MM cells following inhibition of MARCKS phosphorylation [44]. Similar effects were achieved in an MM xenograft model [45].

A key role in bortezomib resistance is played by the increased expression of proteasomes and proteins involved in providing protection from oxidative stress, such as thioredoxin (TXN) [46]. Our findings indicate that higher expression of TXN correlates with shorter PFS. Previous studies have also found TXN to be overexpressed in primary myeloma cells isolated from bortezomib-resistant MM patients, and that overexpression of TXN correlated with poor overall survival in patients with MM [46]. In bortezomib-resistant myeloma cell lines, TXN inhibition overcomes adaptive bortezomib resistance [47]. In addition, higher TXN1 expression levels were found to correlate with myeloma cell survival and growth, and to protect MM cells against increased intrinsic oxidative stress [48]. Moreover, inhibition of TXN1 leads to apoptosis in drug-resistant MM.

Another gene whose high expression significantly correlated with shorter PFS in MM patients is *XBP1*, coding for X-box-binding protein 1. The XBP1 protein is an important transcription factor necessary for differentiation of B cells into plasma cells, being responsible for the final maturation of plasmablasts to plasmocytes and the induction of immunoglobulin secretion [49]. XBP1 is also a particularly important regulator in the UPR mechanism. It is spliced into two isoforms. One isoform, XBPs1s, activates the genes necessary to reduce ER stress and UPR activation after penetration into the cell nucleus. XBP1 may have a significant impact on resistance to bortezomib in MM cells. Low expression of *XBPS1* has been associated with a lack of sensitivity to PI treatment [50]. Two point mutations in the *XBP1* gene have been identified to date [49,51]: the first, XBP1-L167I, is located within the splice site of the *XBP1* gene, and has been shown to prevent the XBP1 mRNA splicing process needed to form the active XBP1s protein, while the second, *XBP1s*-P326R, is located within the transactivation domain of the XBP1s molecule and has no effect on the splicing process. Cells displaying one of the described mutations lose their sensitivity to bortezomib, inducing disease resistance [52].

In conclusion, our results suggest that high expression of *PSMB5* and *CXCR* may serve as predictors of PFS in MM patients treated with bortezomib-based regimens. In addition, high expression of *POMP* and *RPL5* can be useful to predict shorter survival of these patients. However, further studies are needed to determine the role of these factors in effective strategy for improving anti-myeloma therapy.

## 4. Materials and Methods

### 4.1. Patients

The patients were recruited prospectively in our institution (Department of Hematology, Copernicus Memorial Hospital, Lodz, Poland) as a part of a planned marker study. The main exclusion criterion was using bortezomib-based therapy prior to the study. The main inclusion criteria were diagnosis of multiple myeloma according to International Myeloma Working Group (IMWG) criteria and planned treatment with a bortezomib-based regimen [53]. A total of 73 MM patients (43 men and 30 women) treated were included. The mean age of the group was 61.9 ± 10.8 years (range: 38.2 to 83.7 years). Their demographic, clinical, and laboratory details are shown in Table 1. All of the patients received bortezomib treatment as first-line treatment or in progression after previous therapy. The participants were classified as either bortezomib-sensitive or bortezomib-refractory, as previously reported, according to their response to bortezomib-based therapy [12,54]. Response to treatment and relapse/progression events were classified according to the IMWG [55,56].

The bortezomib-sensitive patients demonstrated CR, VGPR, or PR lasting longer than six months following discontinuation of bortezomib-based therapies [56,57,58]. In total, 30 patients were bortezomib refractory and 43 were bortezomib sensitive with no progression for at least six months of treatment discontinuation. The control group consisted of 11 healthy volunteers (six women and five men; mean age 61.9 ± 10.8 years; range: 38.2–83.7 years). The study was conducted according to good clinical and laboratory practice. The experimental protocol was conducted in accordance with the Declaration of Helsinki. All procedures were approved by the local ethical committee (The Ethical Committee of the Medical University of Lodz, No RNN/103/16/KE). Informed consent was obtained from all subjects involved in the study.

### 4.2. Blood Collection

Peripheral blood was collected in PAXgene Blood RNA Tubes (Qiagen, Germantown, MD, USA) from 73 multiple myeloma patients and 11 healthy volunteers and stored frozen at −80 °C. Venous blood samples were collected from MM patients, before treatment with bortezomib-based regimens, most commonly on the first day of the bortezomib administration. In previously treated MM patients, blood was collected at the time of progression, during the qualification process for commencement of a new therapy line.

### 4.3. The Analysis of Gene Expression Using Real-Time PCR

#### 4.3.1. Isolation of Total RNA

Frozen blood samples were thawed on ice and total RNA was isolated from 1.5 mL of blood using the QIAamp RNA Blood Mini Kit (Qiagen) according to the manufacturer’s protocol. The final elution of total RNA was performed using 50 µL of RNase-free water. Total RNA quality was determined using the High Sensitivity RNA Screen Tape on a 2200 TapeStation bioanalyzer (Agilent, Santa Clara, CA, USA). The degradation rate of RNA was determined using RNA integrity number (RIN). Only the samples with RIN > 7 were further analyzed. The quantity of RNA was measured using NanoVue Plus Spectrophotometer (GE Healthcare, Wauwatosa, USA. Directly after isolation, RNA was used for the reverse transcription process.

#### 4.3.2. Reverse Transcription Reaction

The reverse transcription was performed using the high-capacity cDNA reverse transcription kit (Applied Biosystems, ThermoFisher Scientific, Waltham, MA, USA,) according to the manufacturer’s protocol. The total volume of reverse transcription mix was 20 µL per reaction, containing 2 µL RT buffer (10X), 0.8 µL dNTP mixture (100 mM of each dNTP), 2 µL random primers (10X), 1 µL RNase inhibitor (20 U/µL), 1 µL MultiScribe Reverse Transcriptase (50 U/µL), and 10 µL RNA template, whereby the reagent mix was prepared on ice. The thermal profile of the reverse transcription program consisted of 10 min incubation at 25 °C, 120 min at 37 °C, 5 min reverse transcriptase inactivation at 85 °C, and cooling down to 4 °C. Total amount of 100 ng of RNA was used as a sample input per 20 µL of reverse transcription reaction. All reactions were performed in a 96-well SureCycler 8800 thermal cycler (Agilent, Santa Clara, CA, USA). The resulting cDNA was stored at −20 °C.

#### 4.3.3. Selection of Reference Genes

A reference gene provides the internal control of the reaction and allows to determine the absolute and reliable value of the studied gene expression using real-time PCR. In order to normalize the variations in sample input for relative quantitation of gene expression, the selection of endogenous control genes was performed using the TaqMan™ Array Human Endogenous Control (Thermo Fisher Scientific, Waltham, MA, USA).

The analysis was performed for six total RNA samples isolated from whole blood of MM patients, according to the manufacturer’s protocol.

The stability of mRNAs was measured by NormiRazor [59]. This is an integrative tool which implements existing normalization algorithms (geNorm, NormFinder and BestKeeper) in a parallel manner. Three reference genes were selected by NormiRazor and TaqMan™ probes ((Thermo Fisher Scientific, Waltham, MA, USA) ACTB (Assay ID: Hs99999903_m1), RPLP0 (Assay ID: Hs99999902_m1), MT-ATP6 (Assay ID: Hs02596862_g1) and their average expression was used as reference.

#### 4.3.4. Real-Time PCR

The expression of nine genes was analyzed in all samples: *ABCB1*, *CXCR4*, *MAF*, *MARCKS*, *POMP*, *PSMB5*, *RPL5*, *TXN,* and *XBP1*. The analysis was performed using commercially available ready-to-use TaqMan^®^ Assays (Applied Biosystems- Thermo Fisher Scientific, Waltham, MA, USA). These were preloaded with a probe labeled with 6-FAM™ dye (emission spectra at ~517 nm) and forward and reverse primers for the amplification of the following genes: *ABCB1* (Assay ID: Hs00184500_m1), *CXCR4* (Assay ID: Hs00976734_m1), *MAF* (Assay ID: Hs00193519_m1), *MARCKS* (Assay ID: Hs00158993_m1), *POMP* (Assay ID: Hs01106088_m1), *PSMB5* (Assay ID: Hs00605652_m1, *RPL5* (Assay ID: Hs00851991_u1), *TXN* (Hs00828652_m1), *XBP1* (Assay ID: Hs00231936_m1).

The PCR mixture consisted of 10 µL of 2X TaqMan™ Genotyping Master Mix (Aplaied Biosystems-Thermo Fisher Scientific, Waltham, MA, USA), 1 µL of appropriate 20X TaqMan^®^ Assay, and 1 µL of cDNA template. The mixture was filled up with a distilled, DNase- and RNase-free water (Gibco, Gaithersburg, MD, USA) to a final volume of 20 µL. The analysis was carried out using the TOptical thermal cycler (Analytik, Jena, Germany). The reactions were performed under the following conditions: an initial denaturation step at 95 °C for 10 min, followed by 40 amplification cycles of denaturation (95 °C, 15 s), a single annealing and extension step (60 °C for 1 min). Fluorescence signal detection was performed after each cycle. Gene expression analysis was performed for each sample in duplicates. Absolute quantification analysis was performed using qPCR Soft 3.1.15.0 (Analytik, Jena, Germany).

### 4.4. Statistical Analysis

#### 4.4.1. Data Preparation

Data were normalized based on the mean expression of three mRNAs in a given sample (ACTB, RPLP0, MT-ATP6); this has proved to be the most stable normalization factor (according to NormiRazor). The normalized Ct values were calculated as:

Normalized ΔCt = Ct mRNA − (mean Ct of ACTB, RPLP0 and MT-ATP6)

Normalized ΔCt values for all samples and with class assignments were provided as Table S7.

#### 4.4.2. Analysis

Nominal variables were expressed as percentages and analyzed using the Chi-square test with appropriate corrections if needed: the Yates correction for continuity or Fisher’s exact test.

For continuous variables, normally distributed data were tested using a two-sided independent Student’s *t*-test. Continuous variables were presented as mean ± standard deviation (SD) or medians with 25% to 75% values according to the data distribution. Survival analysis was conducted using a Kaplan–Meier estimate with univariate and multivariate Cox’s proportional hazards models, as well as the log-rank test. Cutoff Finder was used to determine the optimal cutpoint for gene expression dichotomization based on the log-rank test minimum *p*-value approach [60]. A procedure based on stratification of a continuous biomarker variable into two groups seems appropriate for use in clinics where most of the decisions are binary. Although such cutoffs are usually based on the mean or median value of the diagnostic factor, they can also be set based on the distribution of the variable or by optimizing the correlation with response to a treatment or outcome. A common problem in biomarker research is overestimating the actual effect when multiple cutoff points are investigated with no correction for multiple testing. The advantage of Cutoff Finder is that it determines the robustness of particular cutoff points and estimates the effect size with confidence intervals [60].

All statistical analyses were conducted using Statistica Version 13.1 (TIBCO, Palo Alto, CA, USA) and R programming language (version 4.0.2). *p* values lower than 0.05 were considered statistically significant. To control the family-wise error rate (FWER), the significant genes were chosen at 5% using Holm’s step-down method. FWER was used to insure a low probability of any false positives among the differentially expressed mRNA.

## 5. Conclusions

The present study examined the mRNA expression of nine genes with a possible influence on bortezomib sensitivity and refractoriness in MM, viz., *ABCB1*, *CXCR4*, *MAF*, *MARCKS*, *POMP*, *PSMB5*, *RPL5*, *TXN,* and *XBP1*. Of these, *RPL5* was down-regulated in MM patients as compared with the normal individuals. *POMP* was significantly up-regulated in MM patients refractory to bortezomib-based treatment. Multivariate analysis found that high expression of *PSMB5* and *CXCR* and autologous stem cell transplantation were the best independent predictors of PFS, and that high expression of *POMP* and *RPL5* were associated with shorter survival. The clinical and biological importance of these findings need further investigation.

## Figures and Tables

**Table 1:** The characteristics of the MM patients treated with bortezomib-based therapy and healthy donors. Data are presented as frequency, percentage (%) unless otherwise specified.

Variable	MM Total	Refractory	Sensitive	Healthy Donors	*p*
Number of patients	73	43	30	11	-
Gender (%) N (%)	M: 43 (58.9) F: 30 (41.1)	M: 25 (58.1) F: 18 (41.9)	M: 18(60.0) F: 12(40.0)	M: 5 (45.5) F: 6 (54.5)	0.69
Age + SD (range)	61.9 ± 10.8 (38.2–83.7)	62.2 ± 11.5 (38.2–83.7)	61.3 ± 9.7 (39.8–81.6)	63.0 ± 6.2 (52.6–74.4)	0.73
Bortezomib regimen:	-	-	-	-	0.18
VCD	58 (79.5)	32 (74.4)	26 (86.7)	-	
VMP	6 (8.2)	5 (11.6)	1 (3.3)	-	
VTD	4 (5.5)	2 (4.7)	2 (6.7)	-	
VD	4 (5.5)	4 (9.3)	0	-	
IsaVRD	1 (1.4)	0	1 (6.7)	-	
Paraprotein–N (%)	-	-	-	-	0.02
IgG	41 (56.2)	28 (65.1)	13 (43.3)		
IgA	17 (23.3)	11 (25.6)	6 (20.0)	-	
LCD	15 (20.5)	4 (9.3)	11 (36.7)	-	
Prior treatment	12 (16.4)	11 (25.6)	1 (3.3)	-	0.01
Bone involvement at diagnosis	40 (54.8)	23 (53.5)	17 (56.6)	-	0.96
Calcium > 2.75 mmol/L at diagnosis	12 (16.4)	7 (16.3)	5 (16.7)	-	0.89
HB < 10g/dL at diagnosis	26 (35.6)	14 (32.6)	12 (40.0)	-	0.73
Creatinine > 2 mg/dL at diagnosis	10 (13.7)	4 (9.3)	6 (20.0)	-	0.31
International Staging System (ISS) at diagnosis	I: 22 (30.1) II: 17 (23.3) III:32(43.8)	I: 14 (32.6) II: 10 (23.3) III: 18(41.9)	I: 8 (26.7) II: 7 (23.3) III: 14(46.7)	-	0.86
CRP > 5 mg/L	33 (45.2)	16 (37.2)	17 (56.7)	-	0.06
Beta2-microglobuline increased (>3mg/L)	51 (69.9)	31 (72.1)	20 (66.7)	-	0.36
LDH > 240U/L	9 (12.3)	5 (11.6)	4 (13.3)	-	0.85
Cytogenetics (%)	*N* = 41	*N* = 24	*N* = 17	-	
t(4;14)	9 (22.0)	7 (29.2)	2 (11.8)		0.26
t(14;16)	0	0	0		-
t(14;20)	0	0	0		-
del(17p)	6 (14.6)	4 (16.7)	2 (11.8)		1.00
amp(1q)	22 (53.7)	12 (50.0)	10 (58.8)		0.75
del(13q)	8 (19.5)	2 (8.3)	6 (35.3)		0.61
t(11; 14)	1 (2.4)	1 (4.2)	0		
del(1p)	2 (4.9)	1 (4.2)	1 (5.9)		1.00
IGH rearrangements	19 (46.3)	12 (50.0)	7 (41.2)		0.71

Abbreviations: CRP—c-reactive protein; IGH—immunoglobulin heavy chain; LCD—light chain disease; IsaVRD—isatuximab, lenalidomide, bortezomib, dexamethasone; LDH—lactate dehydrogenase; MM—multiple myeloma; VCD—bortezomib, cyclophosphamide, dexamethasone; VD—bortezomib and dexamethasone: VMP—bortezomib, melphalan and prednisone; VTD—bortezomib, thalidomide, dexamethasone.

**Table 2:** mRNA expression in multiple myeloma patients and healthy donors. The higher ΔCt value represents the lower expression of gene at mRNA level.

mRNA	ΔCt MM (*N* = 73) mean ± SD	Δ Ct Healthy Donors (*N* = 11) Mean ±SD	FC	*p*-Value	FWER
ABCB1	7.55 ± 0.99	7.12 ± 0.74	0.74	0.1075	0.6451
CXCR4	3.83 ± 0.82	3.56 ± 0.21	0.82	0.0209	0.1669
MAF	7.75 ± 1.08	7.20 ± 0.85	0.68	0.0737	0.5159
MARCKS	5.99 ± 0.83	5.63 ± 0.90	0.78	0.2346	1.0000
POMP	5.17 ± 0.67	5.12 ± 0.39	0.97	0.7541	1.0000
PSMB5	6.96 ± 0.78	6.80 ± 0.59	0.90	0.4341	1.0000
RPL5	2.73 ± 0.81	2.02 ± 0.46	0.61	0.0004	0.0033
TXN	3.43 ± 0.74	3.69 ± 0.66	1.20	0.2508	1.0000
XBP1	3.26 ± 0.92	3.21 ± 0.66	0.96	0.8036	1.0000

Abbreviations: MM—multiple myeloma; FC—fold change; FWER—family-wise error rate.

**Table 3:** mRNA expression in MM patients sensitive and refractory to bortezomib-based chemotherapy. The higher ΔCt value represents the lower expression of gene at mRNA level.

mRNA	ΔCt Refractory (*N* = 43) Mean ± SD	ΔCt Sensitive (*N* = 30) Mean ± SD	FC	*p*-Value	FWER
ABCB1	7.58 ± 1.02	7.50 ± 0.98	0.95	0.7384	1.0000
CXCR4	3.75 ± 0.70	3.95 ± 0.96	1.15	0.3438	1.0000
MAF	7.70 ± 1.12	7.82 ± 1.03	1.09	0.6516	1.0000
MARCKS	5.79 ± 0.70	6.27 ± 0.92	1.40	0.0190	0.1522
POMP	4.94 ± 0.57	5.48 ± 0.67	1.45	0.0007	0.0062
PSMB5	6.84 ± 0.70	7.12 ± 0.87	1.22	0.1421	0.8523
RPL5	2.69 ± 0.87	2.78 ± 0.75	1.06	0.6622	1.0000
TXN	3.35 ± 0.72	3.55 ± 0.77	1.15	0.2676	1.0000
XBP1	3.08 ± 0.84	3.51 ± 0.97	1.35	0.0537	0.3759

Abbreviations: MM—multiple myeloma; FC – fold change; FWER—family-wise error rate.

**Table 4:** Univariate Cox regression analyses for progression-free survival and overall survival.

Variables	PFS					OS				
	Coefficient	*p*-Value	HR	95% CI		Coefficient	*p*-Value	HR	95% CI	
				Lower	Upper				Lower	Upper
ABCB1 expression (high vs. low)	−0.248	0.2716	0.609	0.252	1.474	−0.226	0.2950	0.637	0.273	1.482
CXCR4 expression (high vs. low)	**0.571**	**0.0327**	**3.134**	**1.099**	**8.940**	0.272	0.2865	1.722	0.634	4.679
MAF expression (high vs. low)	0.261	0.1348	1.685	0.850	3.336	0.390	0.2968	2.183	0.504	9.464
MARCKS expression (high vs. low)	**0.594**	**0.0018**	**3.281**	**1.559**	**6.907**	−0.343	0.1115	0.504	0.217	1.172
POMP expression (high vs. low)	**0.409**	**0.0236**	**2.266**	**1.116**	**4.601**	**0.573**	**0.0108**	**3.144**	**1.303**	**7.585**
PSMB5 expression (high vs. low)	**0.476**	**0.0088**	**2.591**	**1.271**	**5.280**	0.348	0.1497	2.004	0.778	5.158
RPL5 expression (high vs. low)	−0.137	0.4206	0.760	0.389	1.483	**0.641**	**0.0035**	**3.607**	**1.526**	**8.524**
TXN expression (high vs. low)	**0.394**	**0.0290**	**2.198**	**1.084**	**4.456**	0.298	0.1683	1.813	0.778	4.228
XBP1 expression (high vs. low)	**0.479**	**0.0099**	**2.605**	**1.259**	**5.389**	0.270	0.2091	1.715	0.739	3.981
Age	0.006	0.7070	1.006	0.975	1.038	0.037	0.1281	1.038	0.989	1.089
ASCT										
No	**Reference**					**Reference**				
Yes	**−0.487**	**0.0089**	**0.378**	**0.182**	**0.783**	**−0.624**	**0.0157**	**0.287**	**0.104**	**0.790**
Bone involvement at diagnosis										
No	Reference					Reference				
Yes	0.303	0.1043	1.832	0.882	3.805	0.309	0.1932	1.856	0.731	4.709
Calcium > 2.75 mmol/L at diagnosis										
No	Reference					Reference				
Yes	0.374	0.0929	2.112	0.883	5.052	−0.089	0.7501	0.837	0.281	2.495
CRP >5 mg/L										
No	Reference					Reference				
Yes	0.101	0.6100	1.224	0.563	2.663	−0.461	0.0637	0.398	0.150	1.054
HB < 10 g/dL at diagnosis										
No	Reference					Reference				
Yes	0.092	0.6243	1.202	0.576	2.505	0.009	0.9698	1.018	0.409	2.530
ISS I	Reference					Reference				
ISS II	−0.682	0.0590	0.375	0.124	1.134	0.030	0.9389	1.828	0.460	7.267
ISS III	0.383	0.1594	1.089	0.509	2.326	0.544	0.0684	3.056	1.035	9.021
Creatinine > 2 mg/dL at diagnosis										
No	Reference					Reference				
Yes	−0.396	0.1952	0.453	0.136	1.502	−0.253	0.4984	0.603	0.140	2.606
LDH >240U/L										
No	Reference					Reference				
Yes	0.188	0.4221	1.457	0.581	3.651	0.411	0.1526	2.277	0.737	7.032
Gender										
F	Reference	0.1008	0.564	0.284	1.118	Reference				
M	−0.287					0.352	0.1583	2.022	0.760	5.376

Abbreviations: ASCT—autologous stem cell transplantation; CRP- c-reactive protein; CXCR-4—C-X-C chemokine receptor type 4; FWER—family-wise error rate: HB—hemoglobin; ISS—International scoring system; LDH—lactate dehydrogenase; MM—multiple myeloma; OS—overall survival; PFS—progression free survival.

**Table 5:** Final multivariate Cox regression analyses for PFS and OS of MM patients.

Variables	PFS				
	Coefficient	*p*-Value	HR	95% CI	
				Lower	Upper
PSMB5 expression (high vs. low)	0.386	0.0451	2.164	1.017	4.603
CXCR expression (high vs. low)	0.748	0.0073	4.465	1.496	13.320
ASCT					
No	Reference				
Yes	−0.612	0.0024	0.294	0.133	0.649
Variables	OS				
POMP expression (high vs. low)	0.523	0.0258	2.849	1.135	7.148
RPL5 expression (high vs. low)	0.664	0.0026	3.777	1.591	8.963

Abbreviations: ASCT—autologous stem cell transplantation; HR—hazard ratio; MM—multiple myeloma; OS—overall survival; PFS—progression free survival.

## Data Availability

Data is contained within the article or supplementary material are available according to “MDPI Research Data Policies” at https://www.mdpi.com/journal/cancers/ instructions#suppmaterials. Normalized ΔCt of mRNA expression for all samples with class assignments are provided in Supplementary Table S7.

## Supplementary Materials

The following are available online at https://www.mdpi.com/2072-6694/13/5/951/s1. Supplementary Table S1: *ABCB1*, *CXCR4*, *MAF*, *MARCKS*, *POMP*, *PSMB5*, *RPL5*, *TXN,* and *XBP1* mRNA expression in MM patients with complete remission (CR) to bortezomib-based chemotherapy and those without. Supplementary Table S2: *ABCB1*, *CXCR4*, *MAF*, *MARCKS*, *POMP*, *PSMB5*, *RPL5*, *TXN,* and *XBP1* mRNA expression in MM patients with at least very good partial response (VGPR), partial response, stable disease, or disease progression (<VGPR) after bortezomib-based treatment. No difference was found between the two groups. Supplementary Table S3: mRNA expression in treatment-naive and previously treated MM patients. A higher ΔCt value represents lower expression of the gene at the mRNA level. Supplementary Table S4: Univariate Cox regression analyses for progression-free survival with missing data (*n* = 7) replaced by overall survival. Supplementary Table S5: Univariate Cox regression analyses for progression-free survival and overall survival- mRNAs expression as continuous variables. Supplementary Table S6: Comparison of final Cox regression of multivariate models based on dichotomized variables (model 1) and continuous variables (model 2). Supplementary Table S7: Normalized ΔCt of mRNA expression for all samples and with class assignments. Supplementary Figure S1: Kaplan–Meier plots for previous treatment and treatment-naïve groups in the univariate analysis for PFS.

## Abbreviations

ABCB1	Adenosine-triphosphate-binding cassette sub-family B member 1
ACTB	beta-actin gene
ASCT	autologous stem cell transplantation
BM	bone marrow
CXCR-4	C-X-C chemokine receptor type 4
DLBCL	diffuse large B-cell lymphoma
ECM	extracellular matrix
FWER	family-wise error rate
IPO8	Importin 8 gene
IsaRVD	isatuximab, lenalidomide, bortezomib, dexamethasone
MAF	musculoaponeurotic fibrosarcoma
MARCKS	myristoylated alanine-rich C-kinase substrate
MM	multiple myeloma
MT-ATP6	mitochondrially Encoded ATP Synthase Membrane Subunit 6 gene
NRF2	nuclear factor erythroid 2-related factor 2
NF-κB	nuclear factor kappa B
OS	overall survival
PC	plasma cells
POMP	proteasome maturation protein
PFS	progression free survival
PI	proteasome inhibitor
PSMB5	proteasome subunit β type 5
RPLP0	Ribosomal Protein Lateral Stalk Subunit P0 gene
RPL5	ribosomal protein L5
UPR	unfolded protein response
TXN	thioredoxin
VCD	bortezomib, cyclophosphamide, dexamethasone
VD	bortezomib and dexamethasone
VMP	bortezomib, melphalan and prednisone.
VTD	bortezomib, thalidomide, dexamethasone
TXN	thioredoxin
XBP1	X-box binding protein 1
