# Supplementary material for: The Prognostic Value of Whole-Blood PSMB5, CXCR4, POMP, and RPL5 mRNA Expression in Patients with Multiple Myeloma Treated with Bortezomib
Source: Cancers (Basel). 2021 Feb 25;13(5):951. doi: 10.3390/cancers13050951 (PMC7956525; doi:10.3390/cancers13050951)
Supplement: Supplementary file 1 [file cancers-13-00951-s001.pdf]

cancers

Supplementary

The prognostic value of whole-blood PSMB5, CXCR4, POMP and RPL5 mRNA expression in patients with multiple myeloma treated with bortezomib

Supplementary Materials

Table S1. mRNA expression in MM patients with complete remission (CR) to bortezomib-based chemotherapy and those without. The higher ΔCt value represents lower expression of the gene at mRNA level.

mRNA	ΔCt <CR mean ± SD	ΔCt CR mean ± SD	FC	*p*-Value	FWER
ABCB1	7.54 ± 1.00	7.56 ± 1.00	1.01	0.9462	1.0000
CXCR4	3.79 ± 0.81	3.92 ± 0.86	1.10	0.5222	1.0000
MAF	7.65 ± 1.12	7.97 ± 0.98	1.25	0.2206	1.0000
MARCKS	5.95 ± 0.73	6.09 ± 1.01	1.10	0.5506	1.0000
POMP	5.10 ± 0.59	5.31 ± 0.79	1.16	0.2610	1.0000
PSMB5	6.97 ± 0.74	6.92 ± 0.88	0.97	0.8123	1.0000
RPL5	2.80 ± 0.76	2.60 ± 0.92	0.87	0.3593	1.0000
TXN	3.36 ± 0.75	3.57 ± 0.70	1.16	0.2532	1.0000
XBP1	3.20 ± 0.92	3.39 ± 0.91	1.14	0.4154	1.0000

Table S2. mRNA expression in MM patients with at least very good partial response (≥VGPR) and with partial response, stable disease or disease progression (<VGPR) after bortezomib-based treatment. Higher ΔCt values represent lower expression of the gene at the mRNA level.

mRNA	ΔCt < VGPR mean ± SD	ΔCt ≥ VGPR mean ± SD	FC	*p*-Value	FWER
ABCB1	7.56 ± 1.04	7.54 ± 0.97	0.99	0.9510	1.0000
CXCR4	3.70 ± 0.79	3.95 ± 0.84	1.19	0.2035	1.0000
MAF	7.63 ± 1.14	7.86 ± 1.02	1.17	0.3754	1.0000
MARCKS	5.91 ± 0.71	6.06 ± 0.93	1.11	0.4558	1.0000
POMP	5.10 ± 0.51	5.23 ± 0.77	1.09	0.4085	1.0000
PSMB5	7.01 ± 0.72	6.91 ± 0.84	0.93	0.6020	1.0000
RPL5	2.74 ± 0.78	2.73 ± 0.85	0.99	0.9552	1.0000
TXN	3.33 ± 0.62	3.52 ± 0.82	1.14	0.2645	1.0000
XBP1	3.30 ± 0.91	3.23 ± 0.93	0.95	0.7327	1.0000

Table S3. mRNA expression in MM patients treatment-naive and previously treated. The higher ΔCt value represents the lower expression of gene at mRNA level.

mRNA	Δ Ct Previously Treated		Δ Ct Newly Diagnosed		*p*
	Mean	SD	Mean	SD	
CXCR4	3.75	0.77	3.81	0.85	0.81
POMP	5.04	0.70	5.18	0.67	0.52
PSMb5	6.96	0.67	6.94	0.81	0.93
RPL5	2.84	0.69	2.73	0.84	0.64
ABCB1	6.95	1.17	7.62	1.02	0.08
MAF	7.48	1.52	7.80	0.99	0.51

MARCKS	6.15	0.73	5.94	0.87	0.40
TXN	3.53	0.53	3.38	0.79	0.42
XBP1	3.12	1.02	3.30	0.89	0.58

Table S4. Univariate Cox regression analyses for progression-free survival with missing data (n=7) replaced by overall survival.

	**Coefficient**	**p-value**	**HR**	**95% CI**	
				**lower**	**upper**
**ABCB1 expression (high vs. low)**	**-0.253**	**0.2240**	0.603	0.267	1.362
CXCR4 expression (high vs. low)	0.524	0.0290	2.851	1.113	7.299
MAF expression (high vs. low)	0.156	0.3242	1.365	0.735	2.533
PSMB5 expression (high vs. low)	0.416	0.0102	2.296	1.218	4.329
RPL5 expression (high vs. low)	-0.089	0.5688	0.836	0.453	1.546
MARCKS expression (high vs. low)	0.464	0.0053	2.532	1.317	4.867
POMP expression (high vs. low)	0.378	0.0249	2.132	1.100	4.129
TXN expression (high vs. low)	0.360	0.0323	2.056	1.063	3.978
XBP1 expression (high vs. low)	0.393	0.0161	2.193	1.157	4.159

Table S5. Univariate Cox regression analyses for progression-free survival and overall survival.

**Variables.**	**PFS**				**OS**			
	**p-value**	**HR**	**95% CI**		***p*-Value**	**HR**	**95% CI**	
			**lower**	**upper**			**lower**	**upper**
**ABCB1 expression**	**0.7519**	**0.947**	**0.674**	1.330	0.5221	0.868	0.562	1.340
CXCR4 expression	0.2101	1.217	0.895	1.656	0.4274	1.211	0.755	1.938
MAF expression	0.1661	1.284	0.902	1.828	0.8496	1.045	0.662	1.650
MARCKS expression	**0.0099**	1.715	1.139	2.591	0.783	0.938	0.595	1.479
POMP expression	**0.0027**	2.660	1.404	5.025	**0.0239**	2.262	1.114	4.587
PSMB5 expression	0.0827	1.435	0.954	2.155	0.298	1.332	0.776	2.283
RPL5 expression	0.7674	1.060	0.720	1.560	**0.0386**	1.592	1.025	2.475
TXN expression	**0.033**	1.653	1.042	2.625	0.1491	1.517	0.861	2.674
XBP1 expression	**0.0353**	1.499	1.029	2.183	0.3182	1.287	0.784	2.114

Table S6. Comparison of final Cox regression of multivariate models based on dichotomized variables (model 1) and continuous variables (model 2).

**Model.**	**Variables**	***p*-Value**	**HR**	95% CI		AIC
				**lower**	**upper**	
**Model 1**	**PSMB5 expression (high vs. low)**	**0.0451**	**2.164**	1.017	4.603	189.486
	CXCR expression (high vs. low)	0.0073	4.465	1.496	13.32	
	ASCT					
	No		Reference			
	Yes	0.0024	0.294	0.133	0.649	
**Model 2**	MARCKS expression	0.0051	0.565	0.378	0.842	194.737
	ASCT					
	No		Reference			
	Yes	0.0022	3.333	1.543	7.194	

Table 7. Normalized ΔCt of mRNA expression for all samples and with class assignments (0- control, 1 –sensitive, 2 –refractory; J13, *fx* -3,61166666666667).

.	ABCB1	CXCR4	MAF	MARCKS	POMP	PSMB5	RPL5	TXN	XBP1
1	8,181667	3,841667	7,331667	6,681667	5,266667	7,141667	2,501667	3,651667	2,991667
2	8,051667	3,416667	7,856667	6,856667	4,961667	7,296667	2,896667	3,666667	3,801667
2	9,858333	4,818333	9,998333	6,218333	5,558333	7,543333	2,883333	3,538333	4,258333
2	7,218333	3,993333	6,818333	6,423333	5,028333	6,818333	1,723333	3,913333	2,858333
2	8,698333	3,783333	8,178333	5,563333	4,593333	6,403333	2,683333	3,003333	3,938333
2	9,908333	2,108333	8,933333	6,438333	5,188333	8,418333	3,348333	2,388333	4,558333
1	6,203333	3,223333	6,118333	6,128333	4,628333	6,168333	2,073333	4,818333	2,228333
1	8,086667	4,026667	8,676667	6,676667	5,101667	7,831667	3,316667	2,676667	4,276667
1	9,303333	5,628333	10,10333	7,768333	6,978333	9,203333	4,578333	4,123333	5,653333
1	6,591667	4,646667	7,661667	6,716667	5,616667	7,076667	2,336667	4,521667	3,821667
1	9,54	5,22	8,725	7,1	5,445	7,49	2,95	2,73	4,445
2	7,626667	3,351667	8,416667	6,496667	5,141667	7,491667	2,541667	3,941667	3,611667
1	8,823333	5,108333	9,498333	6,943333	6,628333	7,903333	3,808333	5,183333	5,293333
1	7,365	4,225	8,885	5,865	5,285	7,675	3,76	2,87	4,09
1	5,893333	3,353333	8,953333	6,188333	5,533333	7,378333	2,003333	3,638333	3,233333
1	7,001667	3,576667	7,566667	5,981667	5,481667	5,766667	1,736667	4,101667	3,566667
2	5,753333	3,178333	6,763333	5,153333	3,848333	5,568333	1,848333	2,283333	2,793333
1	7,658333	0,793333	7,843333	6,133333	5,188333	7,223333	2,578333	2,283333	3,593333
1	7,726667	4,641667	8,396667	7,166667	5,681667	7,821667	3,706667	3,546667	5,011667
1	6,658333	3,368333	6,838333	5,883333	4,993333	7,578333	2,943333	3,248333	3,808333
1	7,053333	3,513333	6,723333	5,263333	4,733333	6,178333	2,268333	3,468333	2,613333
2	6,186667	4,181667	7,111667	6,466667	4,791667	6,586667	2,346667	3,181667	3,371667
1	6,526667	3,631667	7,151667	5,856667	5,156667	6,546667	2,576667	3,241667	3,601667
2	8,67	3,685	9,335	5,505	5,995	7,93	3,74	3,02	3,2
2	6,785	3,24	7,195	4,415	4,62	6,19	2,54	2,67	2,635
1	8,905	4,43	8,665	7,375	6,165	7,65	2,5	3,69	4,395
1	6,383333	3,983333	6,523333	6,458333	4,653333	5,823333	1,928333	3,368333	2,098333
2	6,873333	3,763333	6,613333	5,843333	4,138333	5,898333	1,433333	2,733333	1,523333
2	7,686667	3,911667	7,251667	5,016667	4,646667	6,441667	3,371667	3,271667	2,976667
1	7,605	4,67	7,85	7,495	6,065	7,755	2,765	3,12	3,425
2	6,798333	4,913333	7,268333	6,263333	4,543333	6,158333	2,998333	3,303333	1,928333
2	6,001667	2,851667	3,846667	6,426667	5,086667	6,206667	2,476667	3,916667	1,921667
2	7,745	4,66	9,235	6,72	6,125	7,765	3,445	4,43	3,88
1	7,996667	4,101667	6,731667	6,341667	4,421667	6,271667	1,661667	2,341667	1,851667
1	7,658333	3,473333	7,408333	5,968333	5,288333	5,903333	2,468333	3,398333	3,518333
2	6,665	4,54	8,16	6,28	4,675	6,725	2,355	3,31	4,03
1	6,873333	3,348333	6,703333	4,573333	5,663333	5,913333	2,133333	3,288333	2,223333
2	3,951667	2,351667		6,491667	5,771667		3,081667	2,896667	3,436667
2	8,273333	4,518333	7,758333	6,443333	5,148333	6,608333	2,733333	3,903333	4,253333
1	5,99	4,415	6,565	6,035	4,57	6,065	2,43	3,735	2,265
2	7,258333	4,238333	8,163333	5,518333	5,913333	7,203333	2,898333	4,618333	3,933333
1	7,65	2,42	8,33	4,855	5,845	6,975	3,76	2,65	3,895
1	7,361667	3,571667	7,666667	5,031667	5,226667	6,421667	3,141667	2,976667	2,021667
1	8,47	4,27	8,445	6,005	6,255	8,185	3,6	3,44	4,09
2	7,356667	2,771667	7,416667	5,831667	4,861667	7,256667	3,051667	2,071667	3,006667
2	7,363333	2,908333	7,283333	4,473333	4,863333	7,393333	3,513333	2,433333	3,773333
2	7,19	3,805	9,16	4,78	5,41	7,885	4,135	3,76	3,14
1	8,673333	5,678333	8,968333	8,093333	7,158333	8,578333	3,898333	5,638333	2,748333

2	7,98	4,36	8,485	6,22	5,88	7,735	4,32	4,53	3,715
2	7,293333	2,773333	7,623333	5,363333	4,483333	6,623333	2,123333	2,788333	3,053333
2	7,063333	4,513333	7,023333	5,313333	4,378333	6,548333	2,878333	3,533333	2,833333
2	8,696667	3,841667	9,306667	5,616667	5,071667	7,501667	3,741667	2,816667	3,786667
2	6,613333	4,123333	7,133333	6,968333	5,113333	6,763333	2,098333	3,448333	3,398333
2	6,846667	3,826667	6,496667	6,291667	5,111667	7,036667	3,126667	4,111667	1,481667
2	7,338333	3,228333	7,958333	5,178333	5,208333	5,878333	0,298333	3,303333	1,963333
2	8,965	3,85	7,94	5,585	4,565	6,975	1,48	2,535	3,65
2	6,598333	4,348333	6,788333	6,213333	3,573333	6,058333	1,808333	2,983333	1,748333
2	7,495	3,725	7,3	6,335	5,455	6,63	1,39	4,095	2,775
2	8,203333	4,363333	8,183333	5,963333	5,433333	7,368333	2,888333	3,903333	4,058333
1	7,166667	2,881667	8,306667	4,496667	5,116667	7,266667	2,566667	3,671667	3,446667
2	6,906667	3,176667	6,511667	5,346667	4,441667	6,531667	2,261667	3,761667	1,946667
2	9,646667	4,596667	9,566667	5,031667	3,801667	7,911667	3,801667	2,391667	3,786667
1	8,248333	4,098333	8,783333	5,648333	5,648333	7,823333	3,218333	3,518333	3,638333
2	7,113333	3,173333	7,898333	4,573333	5,118333	6,598333	2,263333	2,768333	2,363333
1	6,663333	3,918333	6,823333	5,803333	5,493333	6,948333	2,163333	3,593333	4,278333
2	6,08	5,02	6,26	6,115	5,285	5,315	4,215	5,275	2,515
2	9,191667	3,656667	8,566667	6,446667	5,336667	6,421667	2,146667	2,931667	3,056667
2	7,946667	3,461667	8,376667	6,216667	4,946667	7,016667	1,711667	3,246667	3,871667
1	6,813333	4,343333	6,403333	7,673333	5,118333	7,153333	2,008333	3,918333	3,298333
2	7,921667	2,286667	7,221667	5,186667	4,521667	6,406667	2,671667	2,486667	2,361667
2	7,701667	3,671667	6,741667	4,391667	4,551667	6,201667	3,841667	3,681667	2,311667
2	8,255	3,185		4,34	4,13	6,19	3,965	1,835	3,715
2	7,31	3,05	7,755	5,765	5,03	6,985	2,465	3,315	2,19
0	8,39	3,68	8,685	5,515	4,76	6,84	1,98	3,15	4,47
0	7,103333	3,808333	6,743333	5,868333	5,178333	7,078333	1,493333	3,813333	3,248333
0	7,06	3,765	7,255	6,59	5,07	7,505	1,325	3,645	3,17
0	5,641667	3,671667	5,721667	5,926667	4,706667	5,351667	1,556667	4,031667	2,076667
0	7,213333	3,353333	7,143333	5,578333	5,448333	7,143333	2,603333	3,273333	3,868333
0	7,093333	3,193333	7,053333	5,788333	5,313333	6,808333	2,123333	3,778333	3,218333
0	7,426667	3,391667	7,671667	4,701667	4,826667	7,076667	1,981667	2,546667	3,631667
0	6,576667	3,406667	5,926667	4,531667	4,656667	6,341667	1,701667	3,561667	2,686667
0	7,913333	3,778333	7,683333	5,278333	5,453333	7,073333	2,588333	3,973333	2,618333
0	7,466667	3,666667	7,851667	7,571667	5,906667	7,191667	2,326667	5,216667	3,471667
0	6,445	3,4	7,515	4,595	5,025	6,35	2,525	3,63	2,805

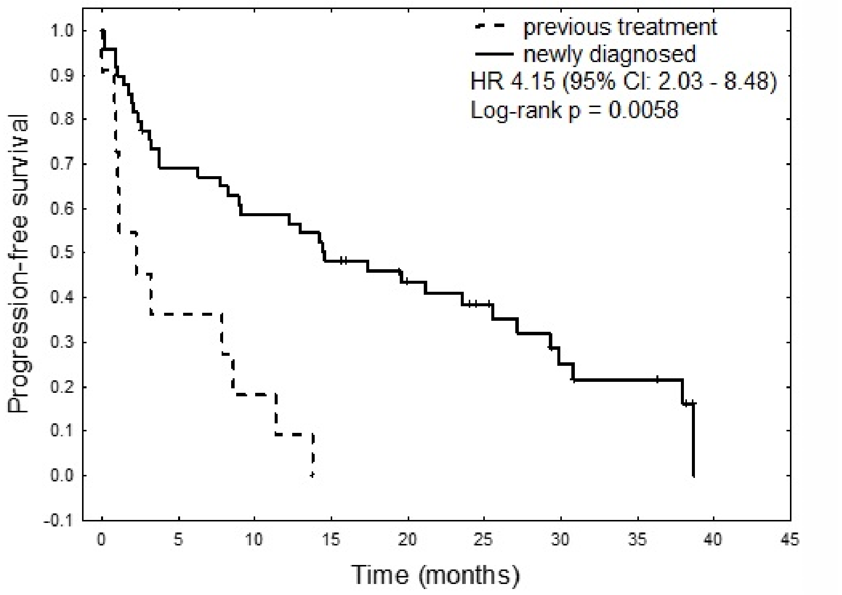

Figure S1. Kaplan-Meier plots for previous treatment and treatment-naïve groups in the univariate analysis fpr PFS.
